# Agminated junctional eruptive nevi following Langerhans cell histiocytosis

**DOI:** 10.1016/j.jdcr.2024.08.026

**Published:** 2024-09-12

**Authors:** Théo Brochet, Ali Dadban, Christophe Attencourt, Catherine Lok, Florian Lombart, Guillaume Chaby

**Affiliations:** a Department of Dermatology, CHU Amiens-Picardie, Amiens, France; b Department of Pathology, CHU Amiens-Picardie, Amiens, France

**Keywords:** eruptive nevi, Langerhans cell histiocytosis, vemurafenib

## Introduction

Multiple melanocytic nevi that appear in a short period are called eruptive nevi (EN). Their number varies from a few up to more than a hundred. They may be distributed on a specific body area, several areas (for instance, sun-exposed areas), or the whole body. They have mostly been described in the setting of congenital blistering disorders, following severe drug eruptions, burns, and exposure to medications.^1^^,^^2^

Implicated medications include cytotoxic, immunosuppressive, and targeted therapies, particularly vemurafenib, in the setting of melanoma or other neoplasms, and melanocyte-stimulating hormone.^2^

When EN occur following a dermatosis or traumatic cause, they appear in clusters on previously affected areas and may be called agminated nevi. They often show clinical and dermoscopic atypias. On the other hand, drug-induced EN are usually diffuse and show no signs of atypia.

Histologically, these nevi are most commonly junctional but may be compound, or dermal. Several reports describe melanomas arising in EN without a history of known melanoma.^2^ Evolution is variable. On follow-up, EN may remain unchanged, increase, or spontaneously disappear.

Langerhans cell histiocytosis (LCH) is a clonal proliferation of histiocytes, which commonly affects the skin. To our knowledge, 9 cases of EN following LCH have been reported. In all of these, LCH was treated by chemotherapy or topical steroids.^3^

We describe the case of a child developing agminated EN 6 years after LCH treated with chemotherapy and vemurafenib.

## Observation

**Fig 1:**
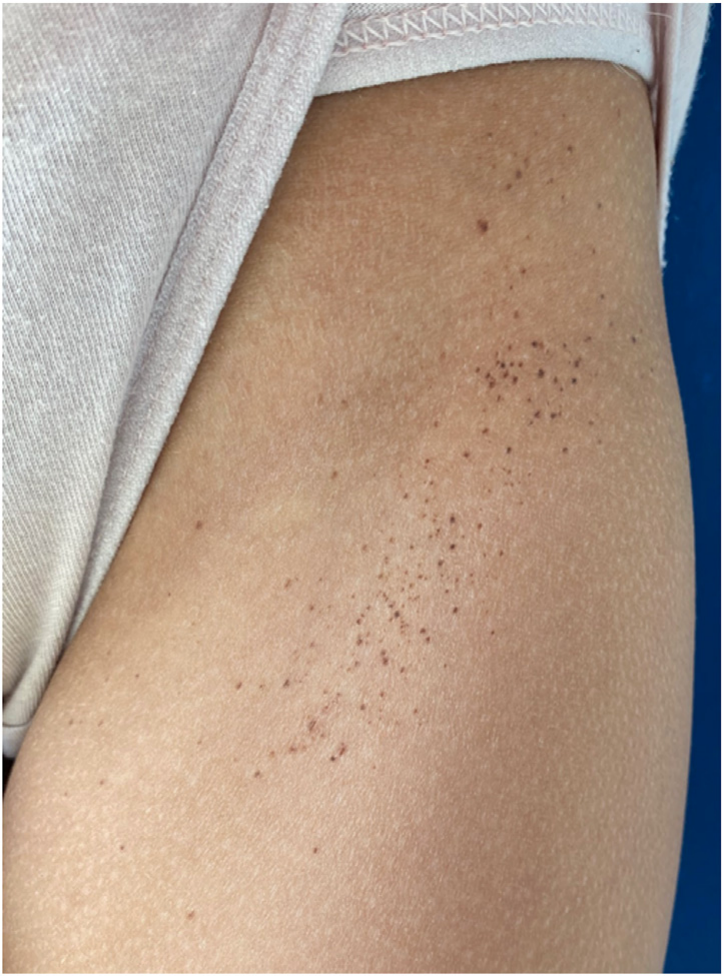
Left inguinal agminated nevi.

**Fig 2:**
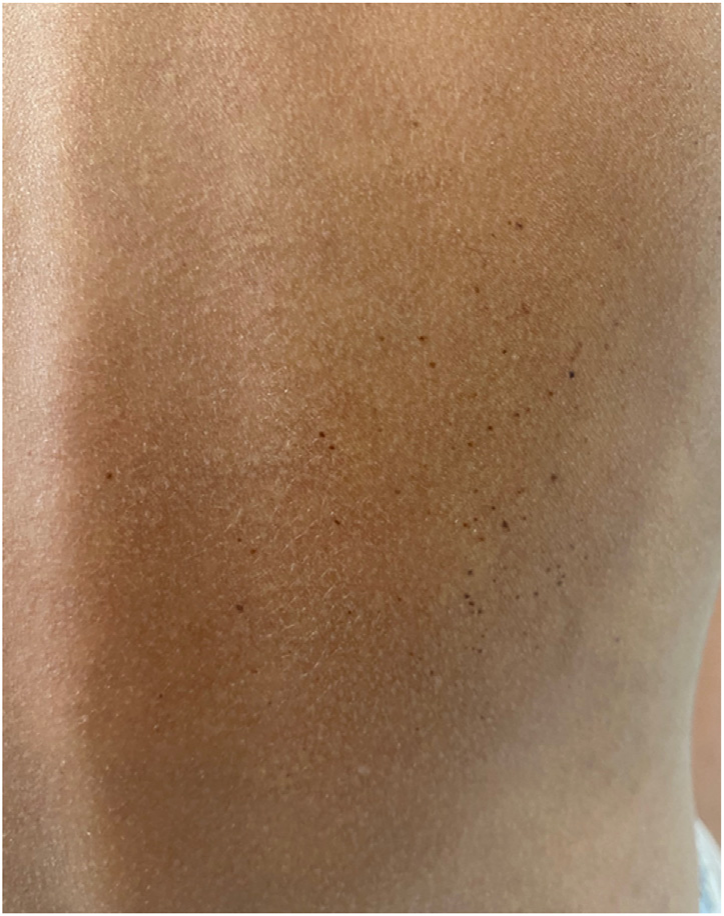
Right dorsal agminated nevi.

A nine-year-old girl presented with multiple asymptomatic pigmented macules appearing over a period of 3 months. Past history was significant for cutaneous and bone LCH, treated at the age of 2 years by vinblastine, cladribine, and prednisone. A relapse was treated by vemurafenib during 6 months up to the age of 3 years because of a somatic BRAF V600 E mutation in tumor cells. The patient achieved complete remission 6 years previously. Physical examination showed more than a hundred symmetrical, 1 to 3 mm, uniformly pigmented macules in clusters of 5 to 10 centimeters on the back and inguinal folds (Figs 1 and 2). Dermoscopy showed a reticular network. The parents confirmed that these lesions were on skin areas previously affected by LCH with the exception of the occipital region, on which we did not find nevi.

**Fig 3:**
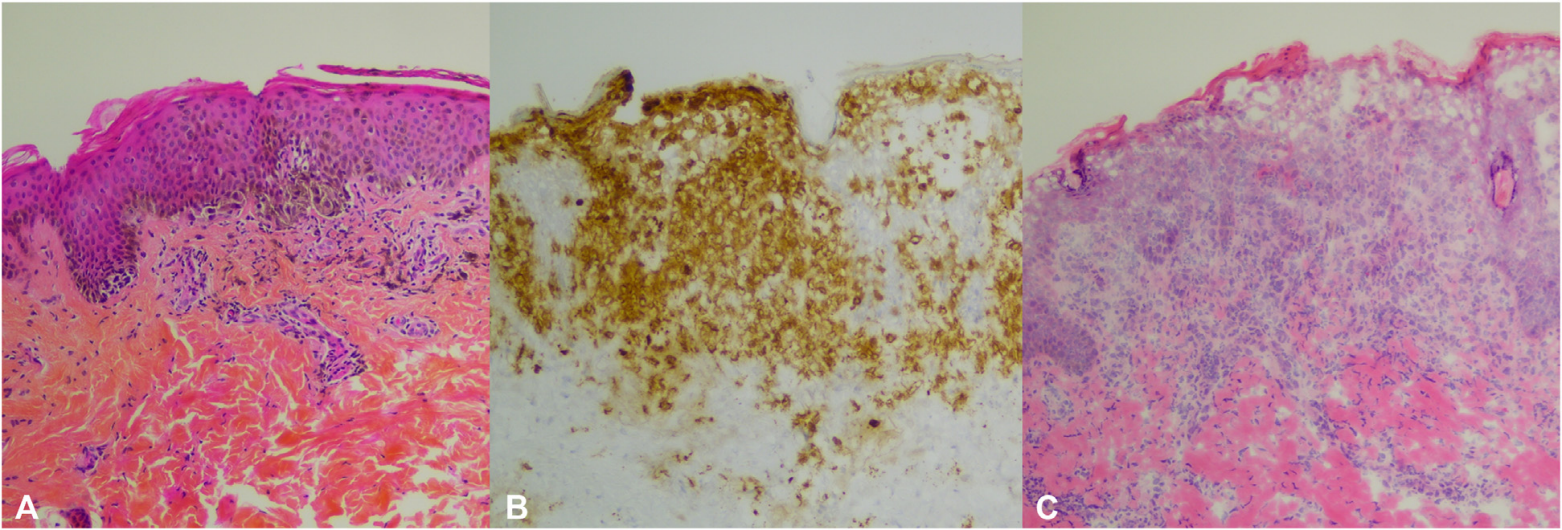
**A,** Junctional nevus: presence of melanocytic theca at the dermal-epidermal junction. **B** and **C,** LCH (frozen sample) proliferation of superficial dermal and intraepidermal CD1a + histiocytic cells. *LCH*, Langerhans cell histiocytosis.

Histology showed nests of melanocytes arranged in a lentiginous pattern along the dermo-epidermal junction without atypia. Immunohistochemistry did not show expression of CD1a and CD68, suggesting no recurrence of histiocytosis (Fig 3). Melanocytes did not express BRAF V600 E in immunohistochemistry.

## Discussion

We summarize the 9 known cases of EN following LCH^3^, ^4^, ^5^, ^6^, ^7^, ^8^, ^9^, ^10^ and add our report in Table I.

**Table I:** Characteristics of described cases of patients with agminated eruptive nevi following Langerhans cell histiocytosis

Features	Age at diagnosis (in years)	Gender	Age at onset (in years)	Localization of nevi	Number of nevi	Histology	Dermoscopy	Treatments of LCH	BRAF expression
Berk et al^4^	7	M	5	Inguinal bilateral	>100 1-2 mm	Spitzoid junctional nevus	NA	Vinblastine	NA
Surinach et al^5^	9	M	2	Inguinal and axillary bilateral	>100 1-4 mm	Spitzoid junctional nevus	Central globules and reticulated periphery	Prednisone and vinblastine	NA
Surinach et al^5^	12	M	11	Inguinal bilateral	>100 Pinhead	NA	Atypical network	Prednisone, vinblastine, and topical nitrogen mustard	NA
Feldstein et al^6^	11	M	8	Inguinal and axillary bilateral and buttocks	>100 3-4 mm	Nevus junctional	NA	Topical triamcinolone and clobetasol	NA
Carreño Gayosso et al^7^	10	F	4	Left inguinal and lumbar	>100 1-2 mm	Dysplastic junctional nevus	Reticulated	Chemotherapy	NA
Rotunno et al^3^	6	F	5	Neck, vulva, inguinal, and axillary bilaterally	>100 2-4 mm	Spitzoid junctional nevus	Reticulated with a few globules	Prednisone, vinblastine, and methotrexate	Negative
Mendonça-Sanches et al^8^	6	M	6	Whole body except head and acral areas	>100 1-4 mm	Junctional nevus	Good network with a few globules	Prednisone and vinblastine Then maintenance therapy with cytarabine and indomethacine	NA
Echavarria et al^9^	7	F	6	Neck, abdomen, inguinal, and axillary bilaterally	>100 1-4 mm	Junctional nevus	NA	Prednisone and vinblastine	Positive
Makino et al^10^	6	F	5	Left inguinal	<100 1-2 mm	Junctional nevus	Globular pattern	Prednisone, vincristine, and cytarabine	NA
This case	9	F	8	Inguinal bilateral and lumbar	>100 1-3 mm	Junctional nevus	Reticulated	Vinblastine, cladribine, and prednisone Then vemurafenib for relapsed LCH	Negative

*LCH*, Langerhans cell histiocytosis; *NA*, not available.

All children developed EN on previous LCH lesions. Interestingly, the head was always spared, in spite of previous LCH involvement. The high presence of eccrine glands, Pacini's corpuscles, or Meissner's corpuscles could increase the formation of nevi by increasing the melanocortin 5 receptor.^1^ Pacini's or Meissner's corpuscles are present in small quantities on the scalp, which may explain the absence of EN on the head. Histology of all of these nevi showed junctional nevi which are more frequent in EN following skin pathologies. Most patients were treated with vinblastine and systemic corticosteroids, but 1 patient was treated with topical treatments only.

Several pathophysiological theories have been proposed.

This may be due to immunotolerance induced by the presence of numerous regulatory T lymphocytes in histiocytosis lesions. The release of pro-inflammatory cytokines by Langerhans cells stimulates nevogenesis.^5^ Some cytokines may increase the expression of the melanocortin 1 receptor, which, when bound to its ligand melanocyte-stimulating hormone alpha, promotes the development of nevi.^2^^,^^6^

Conventional chemotherapies, such as vinblastine, may cause EN because of the immunosuppression they induce, rather than because of their specific therapeutic target.^1^ In fact, reducing immune surveillance may allow nevus growth factors to promote the formation of nevi in predisposed individuals.^1^

As explained above, EN can also occur after treatment with chemotherapies targeted therapies such as vemurafenib.^2^ Inhibiting the BRAF pathway could lead to the activation of alternative survival pathways and the development of EN, according to Perry et al.^1^ The location of EN at sites of LCH damage suggests a pathophysiological involvement of LCH and not just of treatments. Moreover, 1 case has been reported without the use of systemic therapy^6^ and 1 patient developed nevi at the same time as LCH.^10^

## Conclusion

Agminated EN may appear at former sites of LCH, particularly in the inguinal folds. This phenomenon may occur several years after remission and should not raise suspicion of recurrence.

## Conflicts of interest

None disclosed.
